# Paramedics' Newborn Life Support Knowledge and Skills Before and After a Targeted Simulation-Based Educational Intervention

**DOI:** 10.3389/fped.2019.00132

**Published:** 2019-04-10

**Authors:** Lukas P. Mileder, Jennifer Gressl, Berndt Urlesberger, Wolfgang Raith

**Affiliations:** Division of Neonatology, Department of Pediatrics and Adolescent Medicine, Medical University of Graz, Graz, Austria

**Keywords:** resuscitation, infant, newborn, education, simulation, emergency medical technicians

## Abstract

**Objective:** Resuscitation of neonates after birth in the out-of-hospital setting is challenging. Thus, we aimed to assess paramedics' newborn life support knowledge and skills before and after targeted simulation-based training.

**Methods:** Voluntary paramedics were recruited from a single Red Cross division. During a 1-day simulation-based educational intervention, essential aspects of neonatal resuscitation were taught and practiced. Before and after simulation-based training, we assessed (1) knowledge of current European Resuscitation Council (ERC) guidelines using a 20-item-questionnaire and (2) the quality of simulated bag-valve-mask ventilation by measuring face mask leakage, using a respiratory function monitor (Standardized Measurement of Airway Resuscitation Training [SMART], GM Instruments Ltd., United Kingdom).

**Results:** Forty-one paramedics participated in the initial survey and 12 took part in the simulation-based educational intervention. There was a significant increase in the number of correctly answered questions: median 62.1% (IQR 37.5–77.4%) vs. 91.7% (IQR 83.3–100%; *p* = 0.001). A total of 1,332 inflations were analyzed. The incidence of substantial mask leakage >75% decreased significantly after training (15.8 vs. 6.1%; *p* < 0.001), while median mask leakage was similar (17.0% [IQR 0.0–55.0%] vs. 18.0% [IQR 6.0–34.0%]; *p* = 0.414).

**Conclusions:** Among paramedics, theoretical knowledge of current ERC guidelines was moderate in this study. Participation in a targeted simulation-based educational intervention was associated with a significant increase in theoretical knowledge. The initially high incidence of substantial mask leakage >75% was decreased after simulation-based training using respiratory function monitoring.

## Introduction

Neonatal resuscitation requiring positive pressure ventilation is rarely needed in low-risk neonates, with the incidence being as low as 6.8% (1). A Norwegian register study found high perinatal mortality (1.14%) in the sub-group of unplanned out-of-hospital births (incidence 6.8 neonates/1,000 live births) (2). Out-of-hospital neonatal death was most frequently associated with perinatal conditions and sub-optimal postnatal care (3). In a state-based retrospective cohort study, planned out-of-hospital births were also associated with significantly higher perinatal mortality compared to planned in-hospital births (3.9 vs. 1.8 deaths per 1,000 deliveries, *p* = 0.003) (4).

Current European Resuscitation Council (ERC) guidelines recommend that “ideally, two trained professionals should be present at all home deliveries” (5). This recommendation does not only include midwives attending planned home deliveries, but also emergency medical services personnel and emergency physicians. Besides multiple unchangeable factors like the low incidence of out-of-hospital neonatal resuscitation, limited space in the home environment as well as suboptimal medical equipment and resuscitation devices, we recognize some factors including theoretical knowledge of ERC guidelines, high stress levels, and a lack of experience in basic ventilation skills that could be improved by targeted training.

Therefore, the aim of the present study was to assess (i) paramedics' theoretical knowledge of newborn life support, (ii) their practical ventilation skills, and (iii) the effect of targeted simulation-based training. We hypothesized that targeted simulation-based training using respiratory function monitoring would result in significant improvements both in cognitive knowledge and practical (ventilation) skills.

## Materials and Methods

We performed a prospective observational study. Primary outcome was theoretical knowledge of current ERC guidelines (5); secondary outcomes were practical ventilation skills measured by face mask leakage [%] and incidence of substantial mask leakage, defined as >75% (6), during non-invasive bag-valve-mask ventilation of a neonatal manikin. Parameters were assessed before and after a 1-day simulation-based educational intervention.

### Participants

Active paramedics from the Red Cross division in Nestelbach near Graz, Austria, were recruited for the study during an in-house training. Participation was voluntary without financial compensation.

### Assessment of Knowledge

Theoretical knowledge was assessed using a self-composed questionnaire, consisting of a general part asking for demographic information and 20 single-choice questions (for a translated English version see Supplementary Material). Questions referred to all aspects of neonatal resuscitation with a particular focus on out-of-hospital care. Wording was directly taken from the ERC guidelines' official German translation (7). The questionnaire was pretested by two medical students and two physicians of the Medical University of Graz, Austria, for clarity of language and content.

Participating paramedics were presented with the questionnaire between February 24, 2017, and April 8, 2017. They were given a maximum of 30 minutes to answer it individually and under supervision. Cognitive aids were not allowed.

### Assessment of Skills

Quality of bag-valve-mask ventilation was assessed using a respiratory function monitor for training purposes (Standardized Measurement of Airway Resuscitation Training [SMART], GM Instruments Ltd., United Kingdom). This consists of a control unit connected to a flow sensor (F10L screen pneumotachograph, GM Instruments Ltd., United Kingdom) and a term neonatal manikin with leak-free airway and an integrated neonatal test lung (Draeger, Drägerwerk AG & Co. KGaA, Germany). For each inflation, peak inspiratory pressure, air flow, tidal volume and face mask leakage are displayed on a connected personal computer.

For assessment of paramedics' ventilation proficiency, the manikin was placed in supine position on a table with an anti-slip mat. Each participant was asked to “ventilate the neonate for 90 s effectively.” For this, we provided participants with the 500-ml self-inflating ventilation bag (Laerdal Silicone Resuscitator pediatric model, Laerdal Medical, Norway) and the round face mask (Neonatal Face Mask size 1, Laerdal Medical, Norway) that are used at the studied Red Cross division. Neither a PEEP valve nor a manometer was attached to the ventilation bag, while the pop-off valve was activated. No external gas flow was connected to the ventilation bag.

During assessment, participants were neither able to see the computer screen nor received any kind of instruction or feedback. The computer screen was filmed using a digital camera on a tripod to record and later manually transfer data into a Microsoft Excel database, as the first series of the SMART system does not allow direct data storage and extraction. As paramedics underwent assessment on a random basis no identifying information was stored, ensuring confidentiality of data, and anonymity of participants.

### Simulation-Based Training

Training took place on April 8th, 2017, in two seminar rooms at the Red Cross headquarter in Nestelbach near Graz and was executed by the corresponding author for a total duration of seven hours. It began with a 45-minute lecture on neonatal resuscitation according to current ERC guidelines (5). Then, the first group of six people participated in a practical technical skills training on “Initial assessment and airway management” (excluding ventilation training), while the second group underwent ventilation assessment in a separate room as described above. After 30 minutes, groups were switched.

After lunch, the whole group participated in a 60-minute bag-valve-mask ventilation training, during which direct visual feedback from the SMART system was used for deliberate simulator practice. During the subsequent 90-minute algorithm training, realistic out-of-hospital neonatal resuscitation scenarios were practiced in teams of three using a low-fidelity newborn manikin (Newborn Anne, Laerdal Medical, Norway). Finally, all participants were asked to answer the questionnaire again and underwent ventilation skills assessment in the same setting as before.

### Statistical Analysis

All data, including demographic information (age, additional medical training, out-of-hospital experience), questionnaire results and face mask leakage, were saved without names using consecutive numbers and analyzed in pseudo-anonymized form. Data are presented as median (minimum/maximum or interquartile range [IQR]). Analyses were performed using IBM®SPSS Statistics 22 (Wilcoxon-rank sum-/Chi square-test) with a significance level of *p* < 0.05.

## Results

Forty-one out of 68 paramedics (60.3%) took part in the initial survey (median age 24 years [19–74]; m:w = 29:12). Of those, seven (17.1%) had been involved in an out-of-hospital birth. None had practical experience in neonatal resuscitation. Seven paramedics (17.1%) had additional medical education as midwife, nurse, nurse assistant or paramedic with advanced emergency competence, and one (2.4%) was a medical student at the time of assessment. In the initial assessment of knowledge prior to the intervention, a median of 62.1% (IQR 37.5–77.4) of the 20 single-choice questions were answered correctly.

Twelve of the participating 41 paramedics (29.3%) underwent the 1-day simulation-based training and answered the second questionnaire. Two paramedics (16.7%) had experienced an out-of-hospital birth and, again, none had been involved in neonatal resuscitation. One of the 12 paramedics (8.3%) had received training as a nurse assistant or midwife. In the second knowledge assessment, 91.7% (IQR 83.3–100.0) of questions were answered correctly (*p* < 0.001). Highest improvements occurred for questions related to oxygen saturation targets after birth, intervals of heart rate assessment and indication for emergency drugs (Table 1 and Supplementary Material).

**Table 1:** Results of the questionnaire-based knowledge assessment (correct answers for each of the 20 questions) before and after the educational intervention.

**Question number**	**Pre-training**		**Post-training**		**Relative improvement [%]**
	**Absolute**	**Relative [%]**	**Absolute**	**Relative [%]**	
1	31/41	75.6	10/12	83.3	+7.7
2	34/40	85.0	11/11	100	+15.0
3	38/40	95.0	12/12	100	+5.0
4	16/40	40.0	11/12	91.7	+51.7
5	8/41	19.5	0/11	0.0	−19.5
6	15/41	36.6	10/12	83.3	+46.7
7	25/41	61.0	12/12	100	+39.0
8	34/39	87.2	11/11	100	+12.8
9	29/40	72.5	11/12	91.7	+19.2
10	32/39	82.1	12/12	100	+17.9
11	23/40	57.5	11/11	100	+42.5
12	23/41	56.1	11/12	91.7	+35.6
13	3/40	7.5	11/12	91.7	+84.2
14	24/39	61.5	10/11	90.9	+29.4
15	27/36	75.0	11/11	100	+25.0
16	32/41	78.0	6/10	60.0	−18.0
17	25/41	61.0	8/12	66.7	+5.7
18	25/39	64.1	11/12	91.7	+27.6
19	4/41	9.8	10/12	83.3	+73.5
20	7/41	17.1	10/11	90.9	+73.8

**Figure 1:**
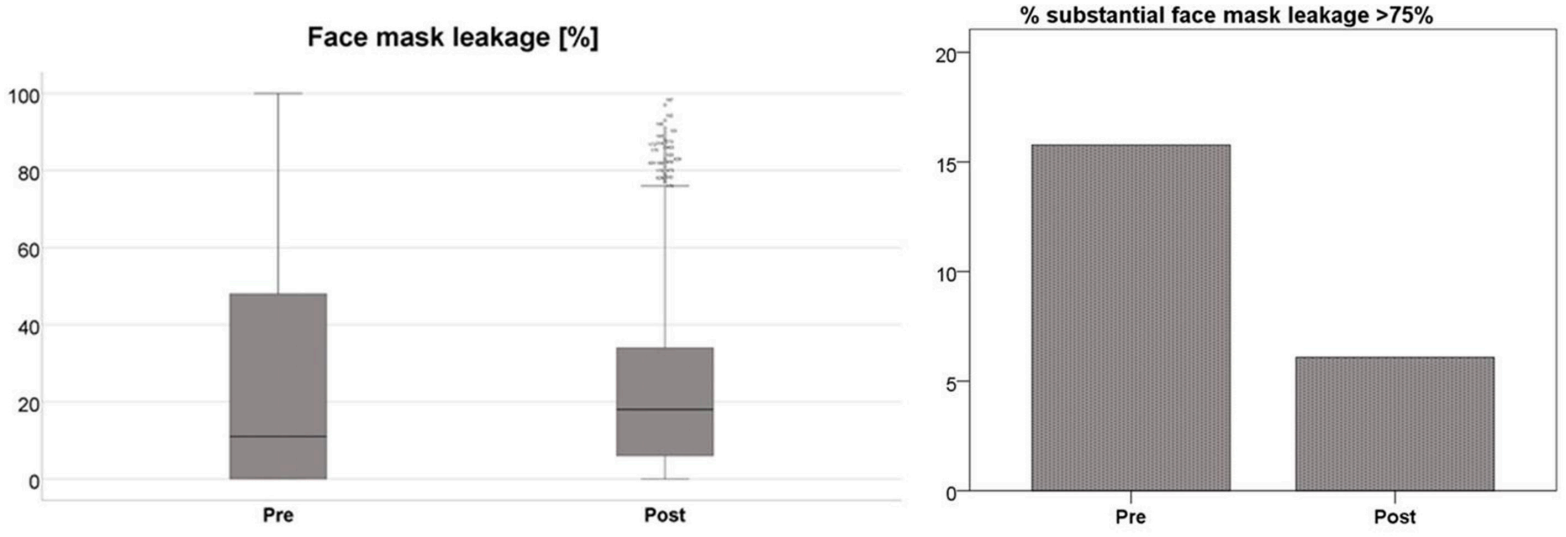
Median face mask leakage [%] before (pre) and after (post) simulation-based training (boxplots, left side; *p* = 0.414) and incidence [%] of substantial face mask leakage >75% (column chart, right side; *p* < 0.001) before (pre) and after (post) simulation-based training.

For assessment of practical (ventilation) skills, we analyzed a total of 1,332 inflations (691 before and 641 after training, respectively). Each participant delivered a median of 55.5 (IQR 48.0–70.0) inflations before and 53 (IQR 48.0–59.0) after training. There was no difference in median face mask leakage when comparing pre- and post-training data: 17.0% (IQR 0.0–55.0%) before vs. 18.0% (IQR 6.0–34.0%) after training (*p* = 0.414; Figure 1). Nevertheless, there was a significant reduction in the incidence of substantial face mask leakage >75% after the educational intervention (median 15.8 vs. 6.1%; *p* < 0.001; Figure 1).

## Discussion

Before training, theoretical knowledge of current ERC guidelines on neonatal resuscitation and transitional support (5) was only moderate in the studied cohort. One possible explanation for this finding may be the rareness of out-of-hospital neonatal resuscitation and the consequent focus on more common medical emergencies during paramedic training. Following targeted simulation-based training, there was a significant increase in the knowledge domain, which is common after simulation-based training (8–10).

The initial quality of simulated ventilation was relatively high, according to the surrogate parameter of median face mask leakage. This is in fact interesting, as none of the paramedics had practical experience in resuscitating neonates after birth. Despite that, participation in the 1-day simulation-based educational intervention including respiratory function monitoring resulted in a reduced incidence of substantial face mask leakage >75% and, thus, improved quality of bag-valve-mask ventilation. This is consistent with other study results (11), with even short training sessions of two minutes resulting in more effective ventilation (12, 13).

Face mask leakage during respiratory support of preterm neonates in the delivery room is a common challenge even for experienced healthcare providers (6, 14). Schmölzer et al. (6) observed a median of 10 (range: 3–117) consecutive inflations with substantial mask leakage >75% delivered to preterm neonates below 32 weeks of gestation in the delivery room, while Murthy et al. (14) found a median mask leakage of 54.5% in 30 preterm neonates with a median gestational age of 30 weeks. Large face mask leakage frequently results in low expiratory tidal volumes, potentially lowering the effectiveness of respiratory support (15). Furthermore, both peak inspiratory pressure and positive end-expiratory pressure are decreased with increasing mask leakage, especially when using a self-inflating bag as in our study (16).

Besides face mask leakage, respiratory parameters such as tidal volume and peak inspiratory pressure also have significant impact on patient outcome. It has been shown that high expiratory tidal volumes (> 6 ml/kg) during postnatal stabilization are associated with development of intraventricular hemorrhage in preterm neonates (17). Barotrauma due to excessive peak inspiratory pressures is one of the contributing factors for the development of chronic lung injury (18). The choice of the ventilation device also contributes to the quality of manual ventilation, as applied tidal volumes and peak inspiratory pressures are higher when using a self-inflating bag compared to a T-piece system (19, 20). Therefore, it would have been of great interest to not only measure face mask leakage in our study, but also delivered tidal volume and peak inspiratory pressure; unfortunately this was not feasible, as these parameters are measured and displayed graphically, but are neither shown numerically on the computer screen nor saved for later analysis in the first version of the SMART system.

By offering quantitative assessment and direct visual feedback, use of a respiratory function monitor can further aid simulation-based manikin training (21). Using respiratory function monitoring does not only lead to improved ventilation quality in the simulated setting (22, 23), but also in the actual healthcare environment (24). Hence, the use of respiratory function monitoring has been recommended for neonatal intensive care (25).

Several studies have shown that skills acquired through simulation-based training can be successfully translated from the simulation laboratory to “real” patient care (26, 27). Hence, it can be assumed that the improved quality of simulated ventilation in our study may lead to improvements in out-of-hospital neonatal resuscitation.

### Strengths and Limitations

We systematically assessed paramedics' knowledge and skills before and after participation in a targeted simulation-based educational intervention consisting of theoretical instruction, part-task training using respiratory function monitoring, and algorithm training with simulated scenarios. This step-wise approach and utilization of proven instructional design features such as range of difficulty, repetitive practice, interactivity, multiple learning strategies, and feedback should allow for an effective learning experience (28). To our knowledge, this is the first study using the novel SMART system both for practice and formal assessment of ventilation skills.

There are some limitations associated with our study. Firstly, in this single center prospective observational study we only assessed paramedics of one Red Cross division, which may limit generalizability of our findings. Generalizability may be further limited by potential differences in neonatal resuscitation training for paramedics in different countries. Secondly, due to the small sample size, we were not able to investigate the potential impact of participants' additional medical training on study results. Thirdly, due to anonymization of questionnaires and practical skills assessments, we were not able to compare individual results before and after the educational intervention. On the other hand, given the comparable range of participants' age and the almost identical experience in out-of-hospital birth and actual neonatal resuscitation, it can be assumed that the group of 12 paramedics who took the educational intervention and who underwent additional testing was a fair representation of the whole group of 41 study participants. At last, we looked at short-term outcome and are, therefore, not able to elaborate on skills maintenance over time.

## Conclusion

Initial newborn life support knowledge among studied paramedics was moderate, while face mask leakage during pre-training assessment was relatively low. We found significant improvements in theoretical knowledge and a decrease in substantial face mask leakage >75% after participation in a targeted simulation-based educational intervention with respiratory function monitoring.

## Author Contributions

LM, JG, BU, and WR made substantial contributions to the conception of the work and interpreted data, revised the manuscript critically for important intellectual content, gave final approval of the version to be published, and agree to be accountable for all aspects of the work in ensuring that questions related to the accuracy or integrity of any part of the work are appropriately investigated and resolved. LM and JG delivered the educational intervention and acquired and analyzed data for the work. LM drafted the manuscript.

### Conflict of Interest Statement

The authors declare that the research was conducted in the absence of any commercial or financial relationships that could be construed as a potential conflict of interest.
