# Supplementary material for: Paramedics' Newborn Life Support Knowledge and Skills Before and After a Targeted Simulation-Based Educational Intervention
Source: Front Pediatr. 2019 Apr 10;7:132. doi: 10.3389/fped.2019.00132 (PMC6467958; doi:10.3389/fped.2019.00132)
Supplement: Supplementary file 1 [file Data_Sheet_1.PDF]

Questionnaire

Demographic data (anonymous):

- Age: ………………………………
- Sex: ○ female ○ male
- Month/year of paramedic training: ………………………………
- Additional medical training:
  - ○ Senior paramedic (without advanced emergency competence)
  - ○ Senior paramedic (with advanced emergency competence)
  - ○ Medical student
  - ○ Nurse
  - ○ Nurse assistant / midwife
  - ○ Other (please specify): ………………………………

- Have you been involved in an out-of-hospital birth during your paramedic work?

  ○ Yes ○ No

- Have you resuscitated a neonate after birth (i.e. delivery of positive pressure ventilation and/or chest compressions) during your paramedic work?

  ○ Yes ○ No

Thank you for your participation in this survey!

The following questions refer to the recommendations by the European Resuscitation Council (ERC) 2015

For each question, only one answer is correct („single choice“):

1) How many trained professionals should ideally be present at all home deliveries?

- ○ At least one
- ○ Two, one of whom must be fully trained and experienced in providing mask ventilation and chest compressions in the newborn
- ○ Three, one of whom must be fully trained and experienced in providing mask ventilation and chest compressions in the newborn
- ○ None

2) How should environment and equipment be prepared before delivery?

- ○ Warm, well-lit, draught free area
- ○ Well-lit area, open windows for delivery of fresh air
- ○ Warm, well-lit area and open windows for delivery of fresh air
- ○ Delivery of fresh air and, if possible, therapeutic hypothermia

3) If a birth takes place in a non-designated delivery area, what is the recommended minimum set of equipment?

- ○ A device for safe assisted lung aeration and subsequent ventilation of an appropriate size for the newborn
- ○ Defibrillator
- ○ Ultrasound device
- ○ Cardiotocography device for recording of fetal heart sounds and uterine contractions

4) In uncompromised neonates who do not require resuscitation after birth, when should the umbilical cord be clamped?

- ○ Immediately
- ○ After at least one minute
- ○ After at least three minutes
- ○ After at least five minutes

5) Which room temperature is recommended for support of transition or resuscitation of neonates after birth?

- 20 – 23 °C
- 23 – 25 °C
- 25 – 28 °C
- More than 28 °C

6) Which three parameters should be simultaneously assessed during initial neonatal assessment?

- Breathing, heart rate, reflexes
- Breathing, heart rate, muscle tone
- Breathing, heart rate, skin color
- Breathing, skin color, muscle tone

7) What do neonates who breathe inadequately or present apneic, with normal or reduced (muscle) tone, and a heart rate of less than 100 min^{-1} require frequently?

- Drying and covering with warm towels, no further interventions
- Drying and covering with warm towels, mask inflation
- Drying and covering with warm towels, mask inflation, and chest compressions
- Drying and covering with warm towels, drug administration

8) Which interventions are first needed in gasping or apneic neonates?

- Chest compressions
- Head tilt and delivery of ventilations
- Opening the airway and delivery of five positive pressure inflations
- Primary endotracheal intubation

9) In which situation should the oropharynx be suctioned?

- Always
- Only if the airway is obstructed
- Only the nasopharynx should be suctioned
- Only if cardiopulmonary resuscitation is required

10) How should the first five positive pressure inflations be delivered?

- Constant initial inflation pressure for 2–3 seconds
- Peak inspiratory pressure should be increased during the first five inflations
- Constant exspiration pressure for 2–3 seconds
- Maximum volume of the ventilation bag should be delivered

11) Which intervention is required if the chest is not rising during positive pressure ventilation?

- Endotracheal intubation
- Auscultation for signs of pneumothorax
- Reposition the baby’s head, 2-person airway control, consider airway alternative, repeat inflation breaths
- Assuring that the baby’s head is tilt, repeat inflation breaths

12) Which initial inspiratory oxygen concentration should be used in term infants?

- 0%
- 21 %
- 79 %
- 100 %

13) At which time point should an oxygen saturation of 90% be reached?

- After one minute of life
- After three minutes of life
- After five minutes of life
- After ten minutes of life

14) What has to be ensured before circulatory support may be effective?

- Room temperature of 25 – 28 °C
- Heart rate below 100 beats min^{-1}
- Successful lung inflation
- Availability of a defibrillator

15) In which situation should chest compressions be delivered?

- During every resuscitation of a neonate
- If no heart rate is detectable
- If the heart rate is less than 60 beats min^{-1} despite adequate ventilation
- If ventilation is not sufficient

16) Which compression-to-ventilation ratio is recommended for resuscitation of a neonate after birth?

- 3:1
- 5:2
- 15:2
- 30:2

17) At which frequency should chest compressions be delivered during resuscitation of a neonate after birth?

- 90 min^{-1}
- 100 min^{-1}
- 120 min^{-1}
- 160 min^{-1}

18) Which compression depth and technique are recommended for delivery of chest compressions during resuscitation of a neonate after birth?

- Compression depth: one half of the anterior-posterior diameter of the chest; Technique: 2-finger-technique
- Compression depth: one third of the anterior-posterior diameter of the chest; Technique: 2-thumbs-technique
- Compression depth: one fourth of the anterior-posterior diameter of the chest; Technique: 2-thumbs-technique
- Compression depth: one third of the anterior-posterior diameter of the chest; Technique: 2-finger-technique

19) How often should the heart rate be re-checked during delivery of ventilation and chest compressions?

- Every 30 seconds
- Every minute
- Every two minutes
- Chest compressions should not be stopped for heart rate re-assessment

20) In which situation should the use of drugs be considered during resuscitation of a neonate after birth?

- In every resuscitation of a neonate
- If the heart rate remains less than 60 beats min^{-1} despite adequate ventilation and chest compressions
- If the heart rate remains less than 100 beats min^{-1} despite adequate ventilation and chest compressions
- In case an emergency physician arrives
